# Crystal structure of *E. coli* PRPP synthetase

**DOI:** 10.1186/s12900-019-0100-4

**Published:** 2019-01-15

**Authors:** Weijie Zhou, Andrew Tsai, Devon A. Dattmore, Devin P. Stives, Iva Chitrakar, Alexis M. D’alessandro, Shiv Patil, Katherine A. Hicks, Jarrod B. French

**Affiliations:** 1 Department of Chemistry, Stony Brook University, Stony Brook, NY 11794 USA; 2 Department of Biochemistry and Cell Biology, Stony Brook University, Stony Brook, NY 11794 USA; 3 Department of Chemistry, SUNY Cortland, Cortland, NY 13045 USA; 4 Half Hollow Hills High School East, Dix Hills, NY 11746 USA

**Keywords:** KPRS, Nucleotide biosynthesis, Purines, Pyrimidines, Ribose-5-phosphate, Phosphoribosyl pyrophosphate

## Abstract

**Background:**

Ribose-phosphate pyrophosphokinase (EC 2.7.6.1) is an enzyme that catalyzes the ATP-dependent conversion of ribose-5-phosphate to phosphoribosyl pyrophosphate. The reaction product is a key precursor for the biosynthesis of purine and pyrimidine nucleotides.

**Results:**

We report the 2.2 Å crystal structure of the *E. coli* ribose-phosphate pyrophosphobinase (EcKPRS). The protein has two type I phosphoribosyltransferase folds, related by 2-fold pseudosymmetry. The propeller-shaped homohexameric structure of KPRS is composed of a trimer of dimers, with the C-terminal domains forming the dimeric blades of the propeller and the N-terminal domains forming the hexameric core. The key, conserved active site residues are well-defined in the structure and positioned appropriately to bind substrates, adenosine monophosphate and ribose-5-phosphate. The allosteric site is also relatively well conserved but, in the EcKPRS structure, several residues from a flexible loop occupy the site where the allosteric modulator, adenosine diphosphate, is predicted to bind. The presence of the loop in the allosteric site may be an additional level of regulation, whereby low affinity molecules are precluded from binding.

**Conclusions:**

Overall, this study details key structural features of an enzyme that catalyzes a critical step in nucleotide metabolism. This work provides a framework for future studies of this important protein and, as nucleotides are critical for viability, may serve as a foundation for the development of novel anti-bacterial drugs.

## Background

The metabolite phosphoribosylpyrophosphate (PRPP) is a building block for the biosynthesis of purine and pyrimidine nucleotides. Ribose-phosphate diphosphokinases or PRPP synthetases (EC 2.7.6.1, also called ribose-phosphate pyrophosphokinases) are a family of enzymes that catalyze the synthesis of PRPP from ATP and ribose-5-phosphate (R5P), yielding AMP as an additional product. This reaction effectively links the pentose phosphate pathway to the nucleotide salvage and de novo biosynthetic pathways as well as the biosynthesis of histidine, tryptophan, and pyridine nucleotide coenzymes [1, 2]. As PRPP is a metabolite that is required at all times in cells, the proper function of PRPP synthetases is essential for life. In humans, mutations that cause overactivity of this enzyme lead to excessive production of uric acid, which causes gout, developmental abnormalities and neurological impairment [3–7]. Three classes (I, II and III) of PRPP synthetases have been described and are differentiated based on phosphate requirements, allosteric regulatory mechanisms, and specificity [8–13]. Several crystal structures have been solved of PRPP synthetases, including those from *B. subtilis* (class I) [8], *M. jannaschii* (Class III), [10], *B. pseudomallei* (class I) [14] and human (class I) [15]. The *E. coli* ribose-phosphate pyrophosphokinase (EcKPRS) is a class I PRPP synthetase with well-defined kinetics [9, 16–18]. Like other class I PRPP synthetases, EcKPRS is known to bind magnesium for efficient catalysis and makes use of an allosteric regulatory site [9, 17, 18]. Here, we report the X-ray crystal structure of EcKPRS, determined to 2.2 Å resolution^1^. The structure, determined by molecular replacement, confirms the hexameric organization of the protein and provides clear molecular details of both the active site and allosteric regulatory site. This work expands the repertoire of structurally characterized PRPP synthetases and will be a valuable tool to aid further detailed analyses of this important protein.

## Results

### Protein expression, purification, crystallization and structure solution

The phosphoribosylpyrophosphate kinase from *E. coli* strain K12 was recombinantly expressed using *E. coli* BL21(DE3), purified used standard immobilized metal affinity chromatography and concentrated to 10 mg/mL for crystallization trials. Initial trials were conducted using sparse matrix screening with the hanging drop vapor diffusion method. After optimization of crystallization conditions, rod-shaped crystals of approximately 200 μm in length grew after 2 weeks. The crystals were frozen in the mother liquor and diffracted to approximately 2.22 Å. The final data set used for refinement was truncated at 2.22 (I/σ = 1.8, CC_1/2_ = 0.0.87, R_merge_ = 0.42, and 95.1% completeness in the highest resolution shell, 2.22–2.26 Å). The space group was *C*222_1_ and there were three molecules in the asymmetric unit with a solvent content of 46% (Matthews number = 2.28). The structure was solved by molecular replacement using the PRPP synthetase from *Burkholderia pseudomallei* (PDB 3DAH) as the search model [14]. After refinement converged, the final KPRS structure had an *R*_work_ (working + test set) of 17.89% and an *R*_free_ of 21.22%. A summary of data collection and refinement statistics is given in Table 1.

**Table 1:** Data collection and processing statistics

Data Collection	
PDB ID	6ASV
Beamline	NE-CAT 24-ID-C
Resolution range (Å)^a^	2.22–83.54 (2.22–2.26)
Wavelength (Å)	0.97910
Space Group	*C*222_1_
Unit Cell Dimensions	
a, b, c (Å)	104.8, 138.4, 137.4
α, β, γ	90, 90, 90
Measured reflections	233,926
Unique reflections	49,727
Mean I/σ	20.5 (1.8)
Completeness (%)	99.3 (95.1)
Redundancy	4.7 (4.4)
R_merge_ (%)	4.7 (42.3)
Data Refinement	
Resolution Range (Å)	2.22–50.0 (2.22–2.26)
Total reflections	47,214
Test set	2485
*R*_work_	17.89
*R*_free_	21.22
No. of protein atoms	6895
No. of phosphate atoms	32
No. of water atoms	298
RMSD from ideal	
Bonds (Å)	0.005
Angles (°)	1.013
Mean B factor (Å^2^)	52.15
Ramachandran	
Favored (%)	96.04
Outliers (%)	0.33
Clashscore^b^	2.66 (100)

^a^Numbers in parentheses correspond to values for the highest resolution shell

^b^Value calculated by MolProbity – value in parentheses corresponds to percentile (100% is best) when compared to a representative set of structures of comparable resolution [30]

### Structure of EcKPRS

**Fig. 1:**
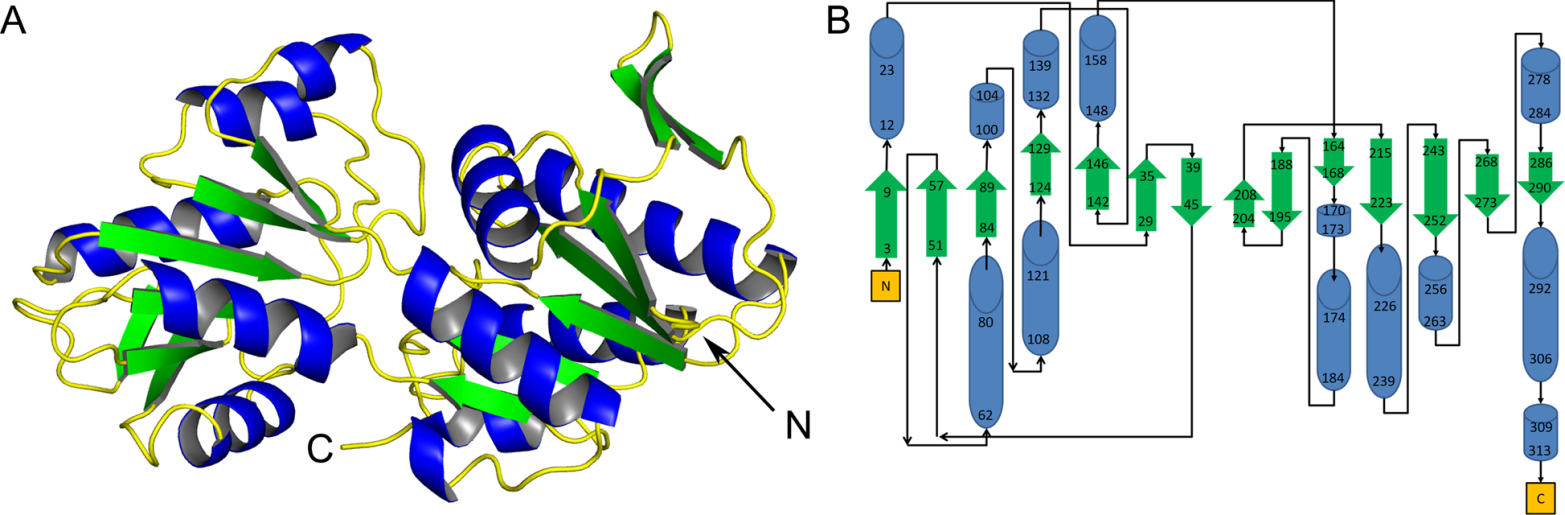
Structure of EcKPRS. The overall fold of the protein (**a**) is shown and the N- and C-termini are labeled (shown is chain A from 6ASV). α-helices are colored blue, β-strands are colored green, and loops are colored yellow. As seen in the structure and in the topology diagram (**b**), EcKPRS has two α/β/α-sandwich domains related by two-fold pseudo-symmetry. Both domains have a type I phosphoribosyltransferase fold

**Fig. 2:**
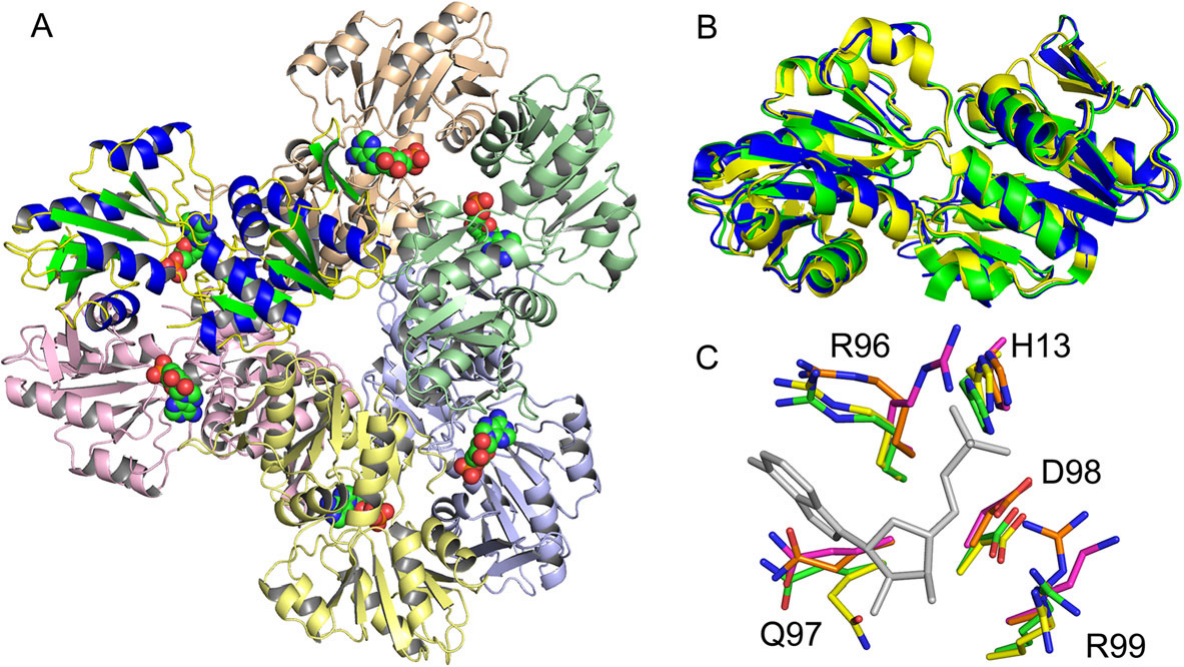
Biological assembly and comparison to other PRPP synthetases. EcKPRS forms a propeller-shaped hexamer with 32 point symmetry (**a**). The location of the active sites is marked by an AMP molecule (shown in space-filling representation; the AMP is from superposition of the EcKPRS structure with that of 3DAH). Note that the protomer colored with blue helices and green strands is in the same orientation as that shown in Fig. 1. **b** The PRPP synthetase from *B. subtilis* (1DKR, green; 0.92 Å RMSD from EcKPRS) and human (2H06, yellow; 0.94 Å RMSD from EcKPRS) superimpose well with the EcKPRS protomer (blue). Comparison of some of the conserved residues (**c**) shows only subtle differences in side chain positions in the active site (EcKPRS shown with green carbon atoms, human PRPP synthetase shown with purple carbon atoms, *B. subtilis* PRPP synthetase shown with orange carbon atoms and the *B. pseudomallei* PRPP synthetase is shown with yellow carbon atoms). For reference, the AMP molecule from the *B. pseudomallei* structure (3DAH) is shown in grey

As observed in other members of this protein family, EcKPRS has two α/β/α-sandwich domains related by two-fold pseudo-symmetry (Fig. 1). These two domains have the type I phosphoribosyltransferase fold and the protein belongs to the ribose phosphate pyrophosphokinase superfamily. The overall quaternary structure of EcKPRS, as observed in the crystal structure (Fig. 2a; composed of the 3 molecules in the asymmetric unit and 3 symmetry related molecules), predicted by the PISA Server [19], and confirmed by size exclusion chromatography (data not shown), is a hexamer. Two unit cells contribute to make the full biologically relevant oligomer. The three molecules in the asymmetric unit are structurally very similar (RMSD between chains A and B = 0.81 Å, between A and C = 0.85 Å, and between B and C = 0.79 Å). The hexamer assumes a propeller-like shape with 32 point symmetry. The C-terminal domain of each protomer dimerizes with one other C-terminal domain to form the blades of the propeller, while the N-terminal domains form the inner core of the structure (Fig. 2a). The inter-subunit interactions in EcKPRS are tight, with the two types of interface (the dimeric interface between two chains forming one blade of the propeller structure, between light purple and yellow chains or between light orange and green chains in Fig. 2a, and the interface between two chains from separate propeller blades, yellow and pink in Fig. 2a) burying approximately 1850 Å^2^ (12.7% of the total surface) and 1600 Å^2^ (11.0% of the total surface) of surface, respectively. The hexamer has a total surface exposed area of 59,260 Å^2^, and a total buried area of 26,980 Å^2^. Note that, in two of the three chains in the unit cell, a loop region spanning residues 196–203 is unstructured and could not be modeled. The corresponding region in the third chain (chain C in the EcKPRS structure), however, had clearly defined density and was well modeled. The three Ramachandran outliers in the structure are from Lys 242 in each of the three chains. The lysine residues are at a surface exposed region of the protein, away from either the active or allosteric sites. These lysine residues were clearly visible in the electron density and have orientations that are conserved in homologous PRPP synthetase structures (3DAH, 64% sequence identity, and 1DKR, 51% sequence identity) [8, 14].

## Discussion

### Comparison of EcKPRS with other PRPP synthetase structures

The Type I phosphoribosyltransferase fold observed in the EcKPRS structure is highly similar to that observed for related phosphoribosyltransferases. The overall structure of the EcKPRS protomer superimposes well to that of the *Burkholderia pseudomallei* ribose-phosphate pyrophosphokinase structure (Fig. 2b; 3DAH, 64% sequence identity to EcKPRS), the *Bacillus subtilis* phosphoribosyl pyrophosphate synthetase (1DKR, 51% sequence identity to EcKPRS), and to the human phosphoribosyl pyrophosphate synthetase (2H06, 48% sequence identity to EcKPRS). The conserved active site residues, including H13, R96, Q97, D98 and R99 (Fig. 2c, numbering from EcKPRS) all adopt similar orientations. The only differences are the slight difference in orientation of the conserved glutamine residue (Fig. 2c, Q97 in EcKPRS) in the active site of *B. pseudomallei* with substrate bound (3DAH) and the arginine residue (R96 in EcKPRS) in the human structure (2H06). The movement of the glutamine residue is likely an induced fit to the AMP binding, leading to more favorable hydrogen bonding contacts between the glutamine and the 3’-OH of the AMP. The alternate conformation of the arginine residue in the human structure is positioned in an unoccupied region in the structure near where the phosphate moiety of the substrate binds. The flexibility of this residue, and its position in the active site, suggests that it too may move upon substrate binding to improve the protein-ligand interactions.

### Active site of KPRS

**Fig. 3:**
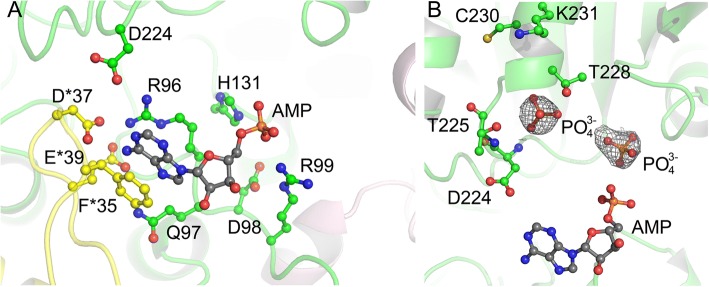
Active site of EcKPRS. The active site of EcKPRS (**a**) is composed of residues from two chains of the protein (one colored with green carbon atoms and the other colored with yellow carbon atoms; oxygen atoms are colored red and nitrogen atoms are colored blue). Interactions with the AMP substrate (AMP has been modeled in from a superposition of EcKPRS to the *Burkholderia pseudomallei* PRPP synthetase (3DAH); the AMP is colored with grey carbon atoms) are made with both chains. In the structure of EcKPRS, two globular regions of density, modeled as phosphates are observed bound in the R5P binding site (**b**). The AMP is shown in the same position as in (**a**) as a point of reference. The electron density shown is from a difference map, generated prior to adding the phosphates, and is contoured at 2.5 σ

The active site of EcKPRS is found between the two domains of the protomer (Fig. 2, AMP modeled in to mark active site position). Each active site, which accommodates the substrates AMP and R5P, is composed of residues from two chains of the hexamer (Fig. 3a). Based upon a superposition of EcKPRS with the *B. pseudomallei* structure, which shows the position of the nucleotide, the conserved active site residues that make contacts with the substrate can be clearly delineated. The purine ring is sandwiched between a phenylalanine residue (F35) from one chain and an arginine (R96) from a second chain (Fig. 3a). Additional polar contacts are made with the ribose and phosphate by residues Gln97, Asp98, Arg92, and His131 from one chain, and Asp37 and Glu39 from the neighboring chain (Fig. 3a). In our maps, we see two approximately globular regions of density consistent in size and shape with phosphate ions. One of these is positioned near where the β- or γ-phosphate of the nucleotide would be expected to be found (Fig. 3b). In addition, a small region of unexplained density is seen, in all three chains, in the position where the nucleotide would bind. While the density is not sufficient to accommodate the full nucleotide structure, it is possible that this density results from partial occupancy or some level of inherent flexibility of the nucleotide in the absence of the R5P co-substrate.

The R5P binding site is known to be made up of residues from the C-terminal region of a single chain. While there are not currently any type-I PRPP synthetase structures available with an R5P ligand bound, the R5P binding site can be inferred from comparison to other phosphoribosyltransferases [20, 21]. The amino acids that make up this active site, Asp224 through Lys231, are contiguously located on a binding loop (Fig. 3b). In the EcKPRS structure, a region of electron density consistent with a phosphate ion, was found to occupy the site believed to bind the phosphate of the R5P substrate, based upon superposition of EcKPRS with the *B. subtilis* and human PRPP synthetase structures [8, 15, 22]. This phosphate is in hydrogen bonding distance with the two backbone amides of Asp224, Thr225 and Gly226 as well as the side chain of Thr225. Placement of the R5P phosphate in this binding pocket would position the ribose ring in close proximity to the phosphates of the nucleotide co-substrate, ideal for efficient catalysis.

### Allosteric site of EcKPRS

**Fig. 4:**
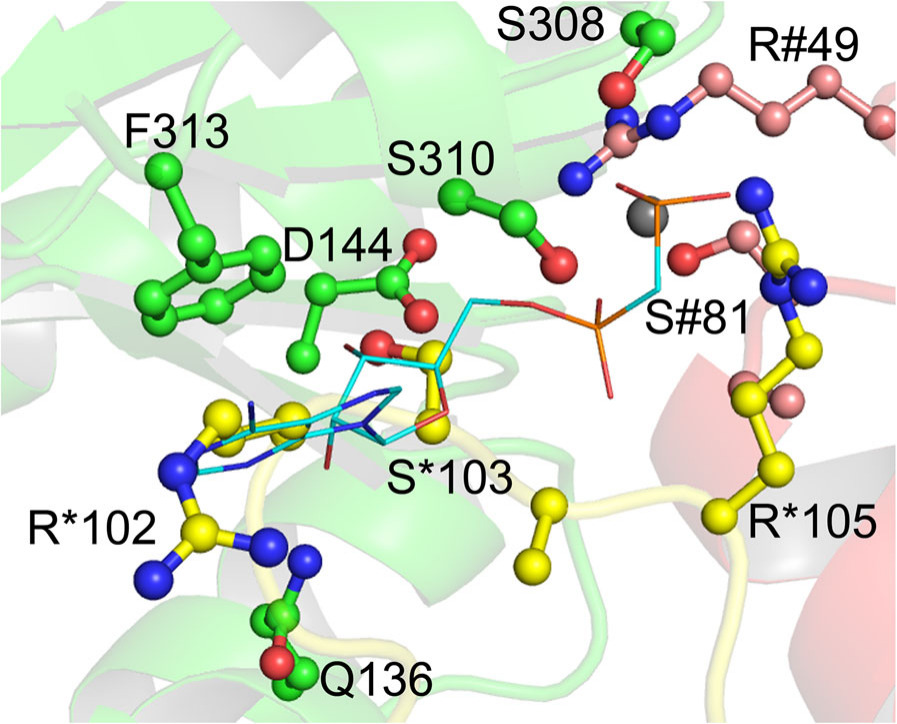
Allosteric site. The allosteric regulatory site of EcKPRS is made up of residues from three different chains of the protein (one chain is colored with green carbon atoms, the second is colored with yellow carbon atoms and numbered with the * symbol, while the third is colored with pink carbon atoms and numbered with the # symbol; oxygen atoms are colored red and nitrogen atoms are colored blue). Binding of the allosteric regulator, ADP (shown here in stick notation with cyan carbon atoms; the position of ADP is modeled from a superposition of EcKPRS with the *B. subtilis* PRPP synthetase structure, 1DKU), is blocked in EcKPRS by the flexible loop containing residues 101 through 105 (yellow carbon atoms)

PRPP synthetases are known to be regulated by ADP and phosphate at an allosteric binding site distal from the active site [15, 17, 22]. Allosteric binding of ADP has been shown to be dependent on binding by Mg^2+^ and phosphate (P_i_) [8, 15, 17, 23]. The allosteric inhibition by ADP appears to be competitive with the activation by Mg^+^ and P_i_, suggesting that they bind to the same site [8, 17, 22]. Several conserved residues make up the allosteric site, which is located at an interface of three chains of the protein [8, 15]. The conserved residues that make up the putative allosteric site in EcKPRS are Gln136, Asp144, Ser308, Ser310, and Phe313 from one chain, Arg49 and Ser81 from a second chain, and several residues (including Arg102, Ser103, and Arg105) on a loop from a third chain (Fig. 4). In the structure of EcKPRS the loop containing Arg102-Arg105 occupies the space where the ADP molecule is purported to bind and an ion (modeled as chloride in EcKPRS) is located in place of where the β-phosphate would be. No other clashes with the protein are evident, suggesting that movement of this loop region would suffice to enable the protein to accommodate the ADP. This is consistent with previous reports that identified this region as a flexible loop [8, 15]. The observed position of the loop also suggests that this part of the structure can occupy the allosteric binding site in place of the native ligand. This suggests that loop binding at this site may act to regulate access by preventing low affinity molecules from binding.

## Conclusions

PRPP synthetase is an important enzyme that produces a key metabolic intermediate necessary for the biosynthesis of purine and pyrimidine nucleotides. Dysfunction of this protein in humans can lead to severe neurodevelopmental disability. Herein, we provide the details of a 2.2 Å X-ray crystal structure of the PRPP synthetase from *E. coli.* Our structure reveals the molecular details of the active site of this hexameric protein and identifies the key residues for substrate binding and catalysis. In addition, the putative allosteric site and flexible loop that defines this region are well resolved in the structure. The flexible loop is found in a position that occupies key binding interactions for the allosteric regulator, thus suggesting a possible mechanism to prevent molecules with low affinity binding to this site. This work expands our understanding of this important class of enzyme and provides a structural framework for future studies.

## Methods

### Protein expression and purification

The *E. coli* (strain K12) ribose-phosphate pyrokinase (KPRS) gene (UniProt P0A717, NCBI NP_415727.1) was synthesized (GenScript) and cloned as an N-terminally His-tagged protein into the pTHT vector (a modified form of pET-28 with a Tobacco Etch Virus protease site in place of the thrombin site [24]) and expressed in *E coli* BL21(DE3) strain. Bacterial cultures were grown in LB broth supplemented with 50 mg/L kanamycin to an optical density at 600 nm of 0.7, at which point protein expression was induced with 0.1 m M isopropyl thiogalactopyranoside (IPTG). The induced cells were grown, with shaking, at 310 K for another four hours. The cultures were pelleted and the resulting pellets were stored at − 20 °C. For purification, freshly thawed cells were resuspended and lysed with lysis buffer (3 mL/g of pellet; 50 mM phosphate pH 7.6, 300 mM NaCl, 10 mM Imidazole). Cell lysates were passed over a pre-equilibrated (lysis buffer) Ni-NTA column (Qiagen). Non-specifically bound proteins were eluted with lysis buffer supplemented with 15 mM imidazole. KPRS protein was eluted using with lysis buffer supplemented with 250 mM imidazole. The protein was buffer exchanged using a 10DG column (BioRad) to storage buffer containing 10 mM Tris-HCl pH 7.6 and 30 mM NaCl, before concentrating the protein to a final concentration of 10 mg/mL as determined by absorbance on a NanoDrop (ThermoFisher). The protein was determined to be greater than 90% pure by SDS-PAGE. The final protein stock was aliquoted, flash frozen in liquid nitrogen, and stored at − 80 °C.

### Crystallization and data collection

Initial sparse matrix screening was conducted for KPRS using the hanging-drop vapor diffusion method at 291 K. The optimized crystallization conditions contained equal volumes of the 10 mg/mL KPRS and a solution consisting of 0.2 M magnesium chloride hexahydrate, 0.1 M HEPES sodium pH 7.5, 30% *w*/*v* Polyethylene glycol 400, and 1 mM ADP. Note that, initial crystal screens were conducted using either 1 mM AMP, 1 mM ADP, or with neither AMP or ADP present. Crystals were briefly soaked in fresh crystallization solution prior to being flash frozen in liquid nitrogen. Data were collected at 100 K at the Advanced Photon Source on the North East Collaborative Access Team beamline 24-ID-C. Data were indexed, processed and scaled using HKL2000 [25]. Data collection statistics are provided in Table 1.

### Data processing, structure solution and refinement

Structures were solved by molecular replacement using MolRep version 11.02.08 [26] with the PRPP synthetase from *Burkholderia pseudomallei* (PDB 3DAH) [14] as the search model. The models were refined using iterative rounds of manual model building with Coot [27], and restrained refinement with Refmac5 [28]. After the refinement had converged, water molecules were added using Coot. The refinement statistics are provided in Table 1.

### Additional software and online tools used

All of the structures shown in figures were made using PyMol version 1.3 (Schrodinger). The topology diagram was derived from results generated by the PDBSum server [29]. The PDBe PISA server was used to determine the surface area of protein-protein interfaces [19]. The MolProbity server [30] was used to validate structures and to generate a clashscore and MolProbity score. All figures were assembled using PhotoShop (Adobe).

## Footnotes

1 Note that, during the preparation of this manuscript, a lower resolution (2.71 Å) structure of *E. coli* KPRS was released in the PDB (4S2U) without a corresponding literature discussion.
